# Activated but functionally impaired memory Tregs are expanded in slow progressors to type 1 diabetes

**DOI:** 10.1007/s00125-021-05595-0

**Published:** 2021-10-28

**Authors:** Joanne Boldison, Anna E. Long, Rachel J. Aitken, Isabel V. Wilson, Clare Megson, Stephanie J. Hanna, F. Susan Wong, Kathleen M. Gillespie

**Affiliations:** 1 Division of Infection & Immunity, School of Medicine, Cardiff University, Cardiff, UK; 2 Present Address: Institute of Biomedical & Clinical Science, University of Exeter, Exeter, UK; 3 Diabetes and Metabolism, Bristol Medical School, University of Bristol, Bristol, UK

**Keywords:** Autoantibodies, CD4^+^ T cells, Regulatory T cells, Slow progression, Type 1 diabetes

## Abstract

**Aims/hypothesis:**

Slow progressors to type 1 diabetes are individuals positive for multiple pancreatic islet autoantibodies who have remained diabetes-free for at least 10 years; regulation of the autoimmune response is understudied in this group. Here, we profile CD4^+^ regulatory T cells (Tregs) in a small but well-characterised cohort of extreme slow progressors with a median age 43 (range 31–72 years), followed up for 18–32 years.

**Methods:**

Peripheral blood samples were obtained from slow progressors (*n* = 8), age- and sex-matched to healthy donors. One participant in this study was identified with a raised HbA_1c_ at the time of assessment and subsequently diagnosed with diabetes; this donor was individually evaluated in the analysis of the data. Peripheral blood mononuclear cells (PBMCs) were isolated, and to assess frequency, phenotype and function of Tregs in donors, multi-parameter flow cytometry and T cell suppression assays were performed. Unsupervised clustering analysis, using FlowSOM and CITRUS (cluster identification, characterization, and regression), was used to evaluate Treg phenotypes.

**Results:**

Unsupervised clustering on memory CD4^+^ T cells from slow progressors showed an increased frequency of activated memory CD4^+^ Tregs, associated with increased expression of glucocorticoid-induced TNFR-related protein (GITR), compared with matched healthy donors. One participant with a raised HbA_1c_ at the time of assessment had a different Treg profile compared with both slow progressors and matched controls. Functional assays demonstrated that Treg-mediated suppression of CD4^+^ effector T cells from slow progressors was significantly impaired, compared with healthy donors. However, effector CD4^+^ T cells from slow progressors were more responsive to Treg suppression compared with healthy donors, demonstrated by increased suppression of CD25 and CD134 expression on effector CD4^+^ T cells.

**Conclusions/interpretations:**

We conclude that activated memory CD4^+^ Tregs from slow progressors are expanded and enriched for GITR expression, highlighting the need for further study of Treg heterogeneity in individuals at risk of developing type 1 diabetes.

**Graphical abstract:**

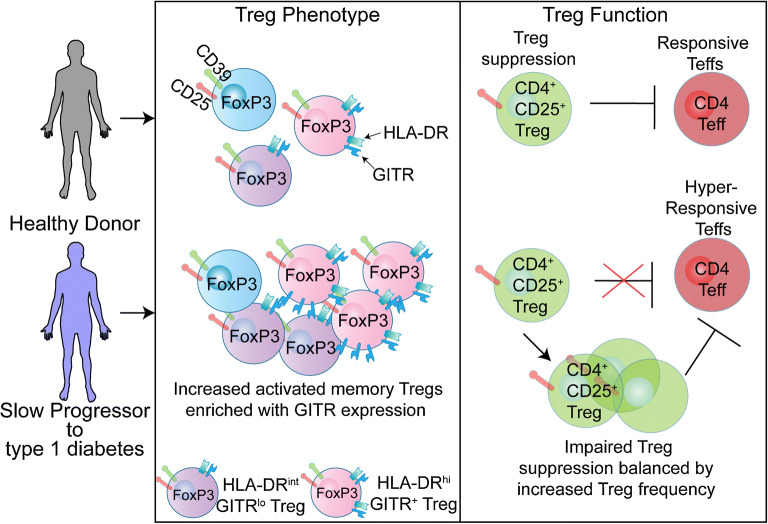

**Supplementary Information:**

The online version contains peer-reviewed but unedited supplementary material available at 10.1007/s00125-021-05595-0.

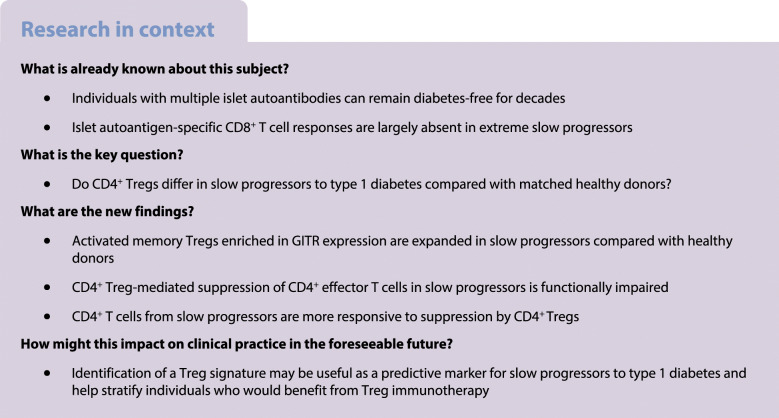



## Introduction

Progression rates from the first appearance of pancreatic islet autoantibodies to development of clinical symptoms of type 1 diabetes are well described in childhood, with 70% of multiple islet autoantibody-positive children developing diabetes within 10 years of seroconversion, which increases to 84% for those followed for 15 years [1]. By contrast, the mechanisms underlying adult-onset type 1 diabetes, which represents more than half of clinical type 1 diabetes, are under-investigated.

A staging system is increasingly widely utilised [2] to define progression to type 1 diabetes; individuals enter stage 1 at development of multiple islet autoantibodies, stage 2 with dysglycaemia, and stage 3 at onset of symptoms. Some multiple islet autoantibody-positive individuals, in stage 1 and 2, progress more slowly and develop adult-onset type 1 diabetes. We previously described a small but very well-characterised group of extreme slow progressors [3] who remained diabetes-free for at least 10 years after the first multiple islet autoantibody sample was detected. Subsequently, we showed that islet autoantigen-specific CD8^+^ T cell responses were largely absent in slow progressors but were readily detectable in individuals with recent-onset and longstanding diabetes [4]. This might suggest that regulation of the autoimmune response is enhanced in these individuals compared with those who progress.

Earlier studies have shown that, although regulatory T cells (Tregs) appear to be normal in number, individuals with diabetes have some functional defects, which include a reduced capacity to respond to IL-2 [5]. In addition, effector CD4^+^ T cells in those who develop diabetes may be more resistant to regulation, demonstrated by a reduction in suppression of effector T cells, by both naturally occurring Tregs and by in vitro generated induced Tregs [6], and diminished IL-2 responsiveness in antigen-experienced CD4^+^ T cells [7].

In the Bart’s Oxford (BOX) study, 14 of 36 (39%) slow progressors developed diabetes during long-term follow-up (median 19 years; IQR 15–26 years). The aim of this study was to examine whether Treg function can be differentiated from age- and sex-matched healthy donors in this well-characterised cohort of extreme slow progressors (including an individual who was diagnosed with diabetes after sampling and 32 years follow-up).

## Methods

### Participants

The BOX study is a longitudinal, population-based study examining risk factors for type 1 diabetes in relatives of individuals diagnosed under the age of 21 years [8]. We previously described the characteristics of long-term slow progressors who remained multiple islet autoantibody positive for more than 10 years but did not develop clinical symptoms of diabetes; the Slow or Non progressive Autoimmunity to the Islets of Langerhans (SNAIL) cohort [3]. Subsequently, ten slow progressors who continued to remain diabetes-free and were willing to provide large-volume blood samples were included in an analysis of T and B cell function [4]. In the current study, eight slow progressors (SP group), with median age 43 (range 31–72 years), had been islet autoantibody positive for between 18 and 32 years (electronic supplementary material [ESM] Table 1). All participants have been at stage 1 of type 1 diabetes progression for significant lengths of time, although some subsequently lost islet autoantibody positivity for some antigens [4]; however one individual has been at stage 2 for at least 6 years but has not developed clinical symptoms (ID: SP 608), and one was diagnosed with diabetes (ID: SP 606). The participant aged 72 years (ID: SP 606) was identified with a raised HbA_1c_ of 53 mmol/mol (7%) at the time the experimental sample was taken. Genetic information for each slow progressor is provided in ESM Table 2, including level of genetic risk. All healthy donors (HD group) were matching within ± 5 years.

### Autoantibodies

Autoantibodies were measured using well-described radioimmunoassays [9]. The results were expressed in units derived from in-house standard curves for insulin autoantibodies (IAA) and zinc transporter 8 autoantibodies (ZnT8A), measured in 5 μl and 2 μl of serum, respectively. For GAD autoantibodies (GADA) and islet antigen-2 autoantibodies (IA-2A), results were derived from a standard curve developed for the NIDDK-sponsored Islet Autoantibody Harmonization Program and were expressed in digestive and kidney (DK) units/ml [10]. These assays were submitted to the Islet Autoantibody Standardisation Programme 2020, where they achieved sensitivity and specificity for GADA of 64% and 97.8%; for IA-2A of 72% and 100%; for IAA of 60% and 97.8%; and for ZnT8A of 68% and 100% (when combining results of variants as described), respectively.

### Peripheral blood samples

Slow progressor and matched healthy donor samples were collected and processed on the same day to avoid any technical batch effects. Peripheral blood mononuclear cells (PBMCs) were isolated from heparinised samples of whole blood via density gradient centrifugation using Lymphoprep (STEMCELL Technologies, Cambridge, UK). 1 × 10^6^ fresh PBMCs were taken for CD4^+^ regulatory T cell flow cytometric analysis. Remaining PBMCs were used for CD4^+^ T cell suppression assays.

### Flow cytometry

Fresh PBMCs were incubated with TruStain (anti-human CD16/32 [Biolegend, UK]) for 10 min at 4°C, followed by fluorochrome-conjugated monoclonal antibodies (mAbs) against cell surface markers for 30 min at 4°C. Multi-parameter flow cytometry was carried out using mAbs (Biolegend): CD19 BV605 (HIB19), CD8 BV605 (HIT8a), CD4^+^ AF700 (A161A1), CD25 PeCy7 (BC96), CD127 PerCPCy5.5 (A019D5), CD45RO BV421 (UCHL1), CD45RA APC-Cy7 (HI100), CD39 PE/DAZZLE 954 (A1), lymphocyte-activation gene 3 (Lag-3) BV786 (11C3C65), CD49b FITC (P1E6-C5), glucocorticoid-induced TNFR-related protein (GITR) BV711 (108–17), HLA-DR BV650 (L243), forkhead box P3 (FOXP3) AF647 (206D), and cytotoxic T-lymphocyte-associated protein 4 (CTLA4) PE (L3D10). Dead cells were excluded from analysis by Live/Dead exclusion dye (Invitrogen, MA, USA). FOXP3 and CTLA4 intracellular staining was performed using the eBioscience nuclear transcription kit. Cells were acquired on LSRFortessa (FACS Diva software, BD Biosciences, UK), and analysis was performed using FlowJo software, version 10.7 (Treestar, USA), and unsupervised clustering methods.

### Unsupervised clustering using FlowSOM and CITRUS

Initial data processing was performed using FlowJo. A total of 10,000 events of live CD19^−^CD8^−^CD4^+^CD45RA^−^ T cells from each donor were down-sampled and imported into R software, version 4.0, and the FlowSOM algorithm was used [11]. Clusters were visualised using both a minimum spanning tree (MST) and t-distributed stochastic neighbour embedding (t-SNE) plots. Heatmaps were generated using R package ggplot2. CITRUS (cluster identification, characterization and regression) was performed using Cytobank [12]. Samples were loaded in Cytobank and traditional gating performed on live CD8^−^CD19^−^CD4^+^CD45RA^−^ cells. Clustering was performed using equal event sampling (8000) and a minimum cluster size set to 1%. A false discovery rate (FDR) was 1%. For both FlowSOM and CITRUS the following markers were included: CD25, CD127, CD39, FOXP3, CTLA4, HLA-DR, GITR, Lag-3 and CD49b.

### CD4^+^ T cell suppression assay

CD4^+^ T cells were isolated by magnetic-activated cell sorting (MACS) using the CD4^+^CD25^+^ T cell isolation kit (Miltenyi), according to the manufacturer’s instructions. Briefly, CD4^+^ T cells, from PBMCs, were negatively isolated. Total CD4^+^ T cells were then separated into CD4^+^CD25^−^ and CD4^+^CD25^+^ fractions by positive selection using anti-CD25 microbeads (CD4^+^CD25^+^ cells; purity >90%). CD4^+^CD25^−^ (4 × 10^5^/well) responder cells were cultured with CD4^+^CD25^+^ at various ratios indicated. All CD4^+^CD25^−^ responder cells were labelled with CFDA-SE (CFSE) (Invitrogen) prior to culture set up. All co-cultures were stimulated with Dynabeads anti-CD3/28 beads (Invitrogen) (0.35 μl beads/4 × 10^5^ cells, 1 bead: 28 responders) and cultured for 3 days before analysis by flow cytometry with CD4 AF700, CD25 PeCy7 and CD134 APC. Dead cells were excluded from analysis by Live/Dead exclusion dye (Invitrogen). All cells were cultured in RMPI complete media containing 2 mmol/l l-glutamine, 100 U/ml penicillin and 10% heat-inactivated AB serum (Sigma-Aldrich, UK). Percentage suppression was calculated by (% CFSE [or CD25/CD134] expression in responder T cell [Tresponder] and Treg co-cultures)/(% CFSE [or CD25/CD134] in Tresponder alone cultures) × 100. Supernatants were taken from co-cultures at the 3-day endpoint, to analyse IFNγ and IL-17A cytokines, which were measured by a Meso Scale Discovery (MSD) (MA, USA) system. MSD was performed according to the manufacturer’s instructions and evaluated using an MSD Sector Imager 6000.

### Statistics

Statistical analyses were performed using GraphPad Prism 8 (GraphPad Software, San Diego, CA, USA). Significance was determined by two-way ANOVA, followed by a Bonferroni post hoc test for Treg suppression assay results, and Wilcoxon matched-pairs signed rank test was performed to compare frequency and surface expression on Treg clusters. Data were considered significant at *p* < 0.05.

### Study approval

The BOX study was approved by the South Central–Oxford C National Research Ethics Committee. The study of slow progressors and control individuals was approved by the South East Wales Research Ethics Committee and conducted in accordance with the principles of good clinical practice established by the International Council for Harmonization/WHO. All participants provided written informed consent prior to enrolment, as mandated by the Declaration of Helsinki.

## Results

### Slow progressors have significantly increased proportions of CD4^+^ effector memory Tregs

We investigated the frequency of Tregs present in the peripheral blood of slow progressors (SP group), compared with age- and sex-matched healthy donors (HD group). We included both CD45RA and CD45RO surface markers to identify resting Tregs and activated Tregs, respectively. Overall, we found no differences in the percentages of CD4^+^CD45RA^+^ naive and CD4^+^CD45RO^+^ memory compartments (ESM Fig. 1a). As Tregs are heterogeneous [13], we chose to employ unsupervised gating using FlowSOM [11]. Surface markers used for analysis included CD25, CD127, FOXP3, HLA-DR, CD39, CTLA4, GITR, CD49b and LAG3, and FlowSOM clustering was performed on down-sampled CD45RA^−^ cells. CD45RA^−^ T cells were chosen because of low or absent expression of the above Treg markers on naive T cells.

**Fig. 1:**
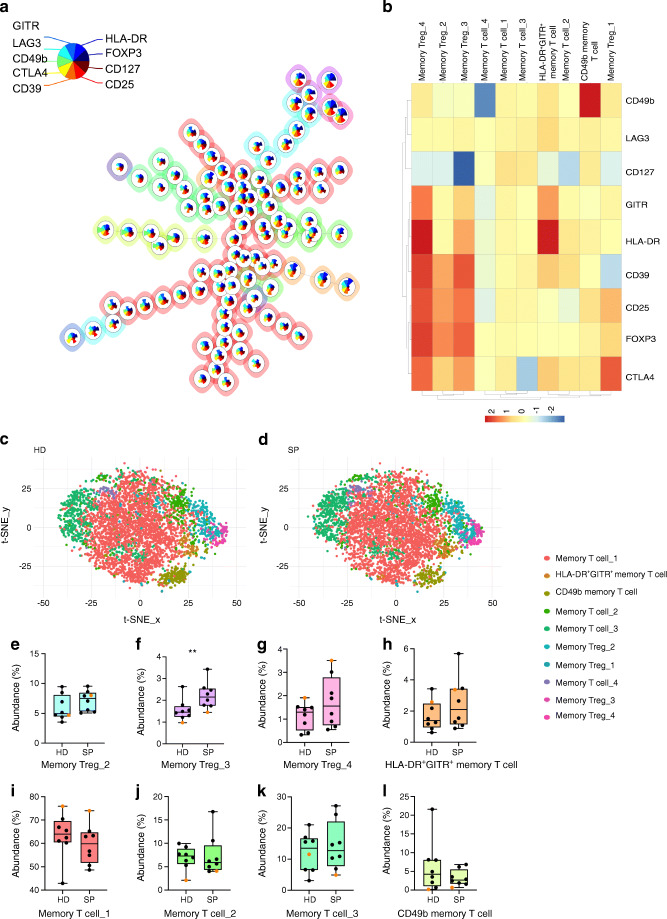
In-depth phenotypical analysis reveals that CD4^+^ Treg subtypes are increased in the slow progressor cohort. Treg compartments generated by FlowSOM, clustering on live CD4^+^CD45RA^−^ cells from all donors. Ten metaclusters were identified based on marker expression: memory T cell_1; memory T cell_2; memory T cell_3; memory T cell_4; CD49b memory T cell; HLA-DR^+^GITR^+^ memory T cell; memory Treg_1; memory Treg_2; memory Treg_3; and memory Treg_4. (**a**) MST of ten metaclusters generated using nine different Treg markers. Each node represents a cluster (100 clusters) and larger metaclusters (10 metaclusters) are coloured around groups of nodes. Pie charts within each node represent expression levels of individual markers. (**b**) Heatmap with each metacluster to show overall marker expression. (**c**, **d**) t-SNE maps generated for the HD group (**c**) and the SP group (**d**) with overlays of each metacluster identified by FlowSOM. (**e**–**l**) Relative abundance boxplots for each metacluster (>0.05% abundance) identified for both HD and SP groups: memory Treg_2 (**e**), memory Treg_3 (**f**), memory Treg_4 (**g**), HLA-DR^+^GITR^+^ memory T cell (**h**), memory T cell_1 (**i**), memory T cell_2 (**j**), memory T cell_3 (**k**) and CD49b memory T cell (**l**). Orange dots represents donor SP 606. **p < 0.01, Wilcoxon matched-pairs signed rank test. The key applies to figure parts (**a**), (**c**), (**d**) and (**e**–**l**)

FlowSOM identified ten distinct clusters (Fig. 1a, MST), which were identified as either a memory Treg or a memory T cell, based on expression of key markers (Fig. 1b, heatmap). CD4^+^ memory T cells and Tregs were each separated into five clusters, characterised by individual phenotypes. Memory Treg clusters 1–4 had an intermediate to high expression of CD25 and FOXP3; however, CD39, HLA-DR and GITR were expressed heterogeneously (Fig. 1b). All metaclusters were expressed in each donor (ESM Fig. 1b) but memory T-cell_4 and memory Treg_1, with a mean abundance of <0.05%, were deemed irrelevant subsets (ESM Fig. 1c). To show the relationship between clusters, t-SNE plots were generated, overlaying each cluster identified for HD (Fig. 1c) and SP cohorts separately (Fig. 1d). t-SNE plots revealed memory Treg subsets 3 and 4 clustered into distinct regions; however, some other subsets such as memory T cell_2 and memory T cell_3 did not form discrete populations and appeared more dispersed, indicating that these smaller subsets are part of a larger population.

Analysis of each cluster from our SP cohort revealed a significant increase in memory Treg_3, compared with the matched HD (*p* < 0.01) (Fig. 1f). Memory Treg_4 and HLA-DR^+^GITR^+^ clusters also increased in individuals in the SP group but did not reach statistical significance (Fig. 1g, h). Donor SP 606 (Fig. 1e–g, orange dots) had an increase in memory Tregs compared with the matched HD participant. Interestingly, we found a modest but not significant decrease in memory T-cell_1 cluster in the SP group compared with the HD group (Fig. 1i), supporting our previous observations [4].

### Predictive modelling confirmed that increased Treg frequency is a signature of slow progressors

**Fig. 2:**
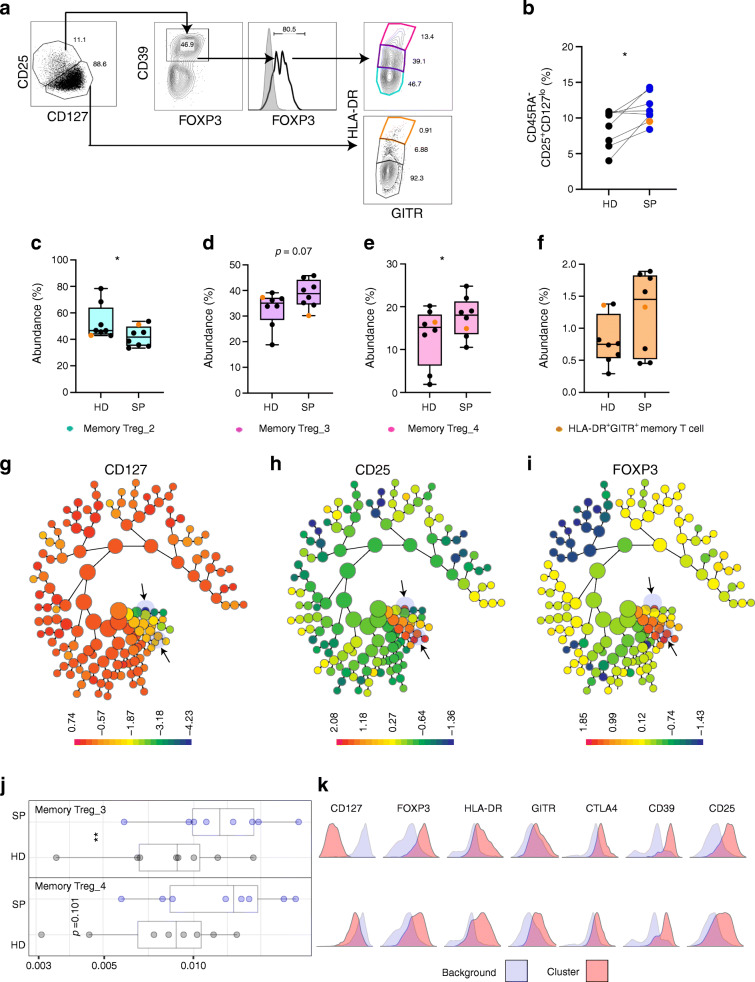
Predictive modelling using CITRUS confirms that increased Treg frequency is a signature of slow progressors. Hierarchical gating (**a**–**f**) and CITRUS analysis (**g**–**k**) was performed comparing SP participants and matched HD participants on CD4^+^CD45RA^−^ T cells. (**a**) Representative plots of parent CD25^+^CD127^lo^ Tregs and CD25^lo^CD127^+^ T cell populations. CD25^+^CD127^lo^ Tregs were then enriched for CD39 and FOXP3, followed by separation by HLA-DR and GITR expression, emulating FlowSOM populations. (**b**) Summary graph of CD25^+^CD127^lo^ frequency in HD (black dots) and SP (blue dots) groups and donor SP 606 (orange). (**c**–**f**) Boxplots for each CD25^+^CD127^lo^CD39^+^FOXP3^+^ and CD25^lo^CD127^+^ gated subset; (**c**) HLA-DR^lo^GITR^−^ (memory Treg_2), (**d**) HLA-DR^int^GITR^lo^ (memory Treg_3), (**e**) HLA-DR^hi^GITR^+^ (memory Treg_4), (**f**) HLA-DR^hi^GITR^+^ (HLA-DR^+^GITR^+^ memory T cell). (**g**–**i**) CITRUS cluster spirals coloured by (**g**) CD127, (**h**) CD25 and (**i**) FOXP3 expression intensity, with clusters identified as different between the SP and HD cohorts highlighted by arrows. (**j**) Boxplots to show relative abundance (proportion) of CITRUS memory Treg_3 and memory Treg_4 in the SP (blue dots) and HD (grey dots) groups. (**k**) Histograms show phenotype of each cluster (pink) and relative expression of Treg markers compared with background expression (blue); upper row, memory Treg_3; lower row, memory Treg_4. Background relates to expression of markers in all other clusters. *p < 0.05, **p < 0.01, Wilcoxon matched-pairs signed rank test

Following our FlowSOM analysis, we confirmed our observations with conventional hierarchical gating (Fig. 2a–f) and CITRUS (Fig. 2g–i). Using hierarchical gating, we emulated our Treg FlowSOM subsets, which were largely based on HLA-DR and GITR expression (Fig. 2a). We revealed that six out of seven in the SP group, including SP 606 (orange dot), had increased CD4^+^CD45RA^−^CD25^+^CD127^lo^ effector memory Tregs, and this was significant (*p* = 0.023) when compared with the HD counterparts (Fig. 2b). Further gating on CD39, followed by FOXP3 expression (mean expression 90.71% [HD] and 89.01% [SP]), we observed increases in memory Treg_3 (HLA-DR^int^GITR^lo^) (*p* = 0.07) and memory Treg_4 (HLA-DR^hi^GITR^+^) subsets (*p* < 0.05) (Fig. 2d,e) in the SP cohort; however, an overall increase in Tregs in donor SP 606 (orange) was not evident. The frequency of Treg populations (of interest) from both FlowSOM (Fig. 1) and hierarchical gating (Fig. 2a–f) expressed as ratios for each individual SP participant normalised to the matched HD participant is shown in ESM Fig. 1d. No difference in CD4^+^CD45RA^+^CD25^+^CD127^lo^ resting Tregs between groups was observed (ESM Fig. 1e). Furthermore, consistent with previous reports [14], CD25^+^ Tregs increased with age in our HD cohort (data not shown).

CITRUS is an algorithm that identifies different cell signatures between grouped data [15] and provides predictive modelling. Here, pre-gating was performed in Cytobank and clustered with the same cellular markers as our FlowSOM analysis. CITRUS analysis provides a visualisation tree (Fig. 2g–i). Here, SP and HD groups are clustered separately, based on marker expression; CD127 (Fig. 2g), CD25 (Fig. 2h) and FOXP3 (Fig. 2i) were used as an example to illustrate clustering of CD4^+^ memory Tregs. Comparing our cohorts, CITRUS identified two distinct clusters that were different in frequency (Fig. 2j). Analysis of cluster phenotypes (Fig. 2k) revealed a similar phenotype to memory Treg_3 and memory Treg_4 metaclusters (FlowSOM; Fig. 1). Overall, high-dimensional immunophenotyping confirmed that slow progressors have an expanded population of CD45RA^−^CD25^+^CD127^lo^ Tregs, which were specifically enriched for activated memory Tregs expressing FOXP3, CD39, CTLA4, HLA-DR and GITR.

### Increased expression of GITR on an activated memory Treg population in slow progressors

**Fig. 3:**
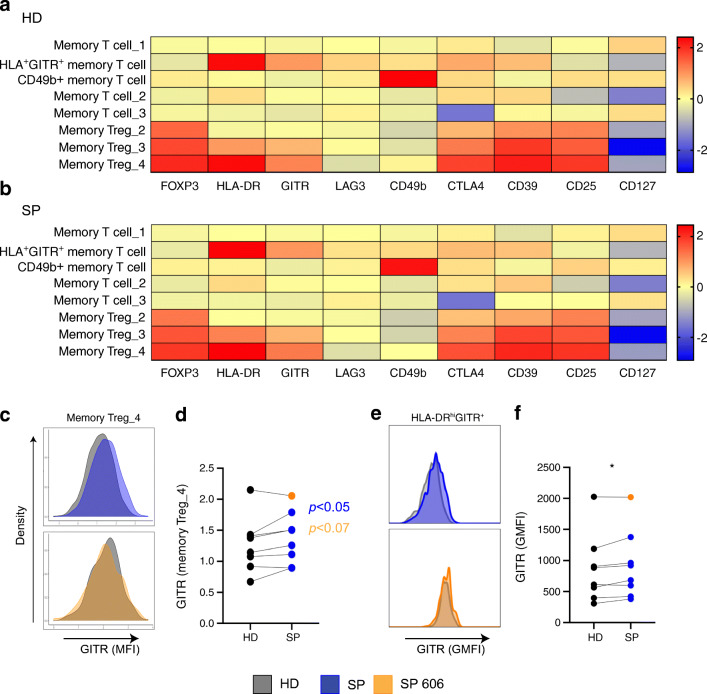
Increased GITR on memory Tregs in slow progressors compared with healthy donors. Each memory CD4^+^ T cell metacluster (memory T cell_1; memory T cell_2; memory T cell_3; CD49b^+^ memory T cell; HLA-DR^+^GITR^+^ memory T cell; memory Treg_2; memory Treg_3; memory Treg_4), from FlowSOM, was examined for a change in each expression marker. (**a**, **b**) Expression heatmaps from the HD (**a**) and SP (**b**) cohorts (SP 606 is not included). (**c**, **d**) FlowSOM GITR expression concatenated from all HD (grey), all SP (blue) and SP 606 (orange) in the memory Treg_4 metacluster, showing histograms (**c**) and summary graph (**d**). Wilcoxon matched-pairs signed rank test, *p* < 0.07 (orange) all donors included, *p* < 0.05 (blue) donor SP 606 and matched HD not included in test. (**e**, **f**) GITR expression, from hierarchical gating, concatenated from all HD (grey), all SP (blue) and SP 606 (orange) in HLA-DR^hi^GITR^+^ Tregs showing histograms (**e**) and summary graph (**f**) *p < 0.05, Wilcoxon matched-pairs signed rank test

Next, we sought to determine if the SP memory T cell metaclusters had different levels of expression of Treg markers. Figure 3a,b shows expression heatmaps, with each marker used for clustering on the eight individual metaclusters, for both SP and HD cohorts, not including donor SP 606. Overall, the heatmaps revealed no striking differences in expression levels for each metacluster in both groups; however, quantitative analysis demonstrated a significant increase in GITR expression in the activated memory Treg_4 metacluster (Fig. 3c,d). Interestingly, we did not observe this GITR increase in donor SP 606, compared with the matched HD donor (Fig. 3c,d, orange). To confirm our observations, we analysed the expression of GITR on the HLA-DR^hi^GITR^+^ population defined by hierarchical gating. Similarly, we observed a significant increase in GITR expression on this memory Treg population (Fig. 3e,f). Gating on all CD39^+^FOXP3^+^ cells also revealed a significant increase of GITR expression (data not shown). Overall, differences in expression of other markers were not observed; however, HLA-DR expression was modestly increased in selected SP donors (ESM Fig. 2a), but this was not confirmed in all donors with hierarchical gating (ESM Fig. 2b). Expression for both GITR and HLA-DR in memory Treg subsets in all donors, normalised to the HD cohort, is shown in ESM Fig. 2c.

### CD4^+^ Tregs from slow progressors show impaired suppressive capacity

To test the functionality of CD4^+^CD25^+^ Tregs in our SP cohort we utilised a well-established in vitro T cell suppression assay [16]. Treg-mediated suppression of CD4^+^ responder T cells (CD4^+^CD25^−^ T cells) was investigated in the SP and HD cohorts by proliferation and activation assays. CD4^+^ responder T cells were labelled with CFSE, co-cultured with varying ratios of CD4^+^ Tregs and stimulated with anti-CD3/28 activation beads. For representative flow cytometric plots, see ESM Fig. 3a.

**Fig. 4:**
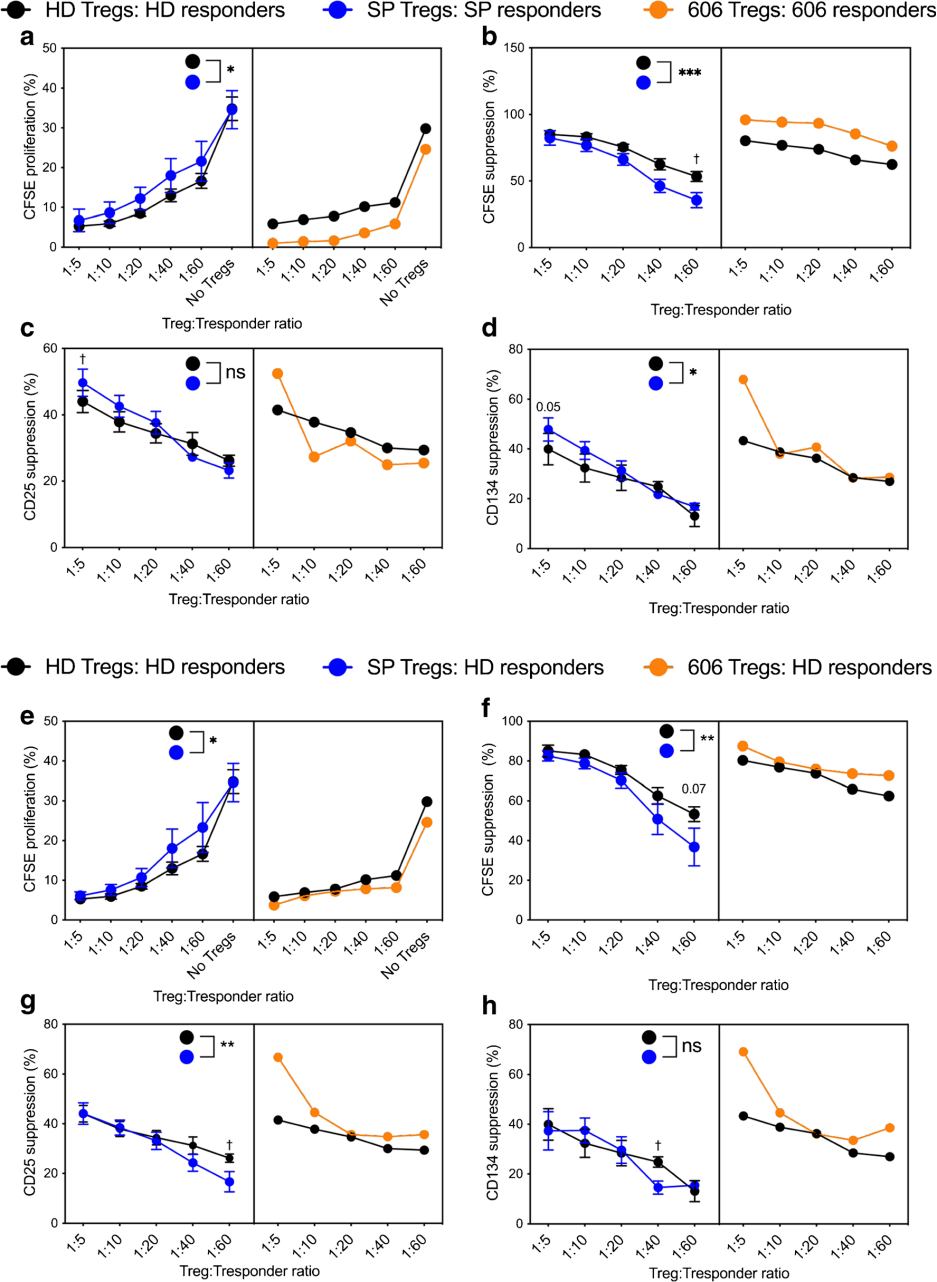
CD4^+^ Tregs from slow progressors have a reduced capacity to control effector CD4^+^ T cells. The SP cohort is shown with blue lines and dots, the HD cohort with black lines and dots, and the orange line/dots represent donor SP 606. CD4^+^ T cells were sorted using a CD4^+^CD25^+^ Treg sorting kit. CD4^+^CD25^−^ (responders) were CFSE-labelled and Tregs were titrated at the observed ratios. Cells were activated with anti-CD3/28 beads and cultured for 3 days before flow cytometry. (**a**–**d**) Tregs cultured with their CD4^+^ Tresponder counterparts (autologous) (**e**–**h**) HD, SP or SP 606 Tregs cultured with HD responders. (**a**, **b** and **e**, **f**) CFSE proliferation (**a**, **e**) and percentage CFSE suppression (**b**, **f**). (**c**, **d** and **g**, **h**) Percentage suppression of CD25 (**c**, **g**) and CD134 (**d**, **h**). Percentage suppression was calculated using the positive control (activated responder cells with no Tregs). *p < 0.05, **p < 0.01, ***p < 0.001, repeated measures two-way ANOVA. ^†^*p* < 0.05 Bonferroni’s post hoc multiple comparisons test

First, we investigated autologous co-cultures to assess overall differences in Treg functionality (Fig. 4a–d). Treg-mediated suppression of CD4^+^ responders, as calculated by percentage of proliferating Tresponder cells in co-cultures divided by Tresponders alone, was significantly decreased in SP participants compared with HD participants (*p* < 0.001) (Fig. 4b). Calculation of the division index corroborated the analysis shown in Fig. 4 using the percentage of cells divided gate (data not shown). However, increased suppression of CD4^+^ T cell activation, measured by both CD25 and CD134, was observed at the higher Treg:Tresponder ratios (Fig. 4c,d). Interestingly, Tregs from SP 606 (orange line) suppressed CD4^+^ responders considerably more than the matched HD. Expression ratios for proliferation and activation markers in individual SP participants, compared with the matched HD participants, are shown in ESM Fig. 3b, demonstrating that the differences are the result of an overall pattern. No change was observed in the levels of activation via anti-CD3/CD28 beads (CFSE proliferation, CD25, CD134 and proinflammatory cytokines), in the absence of Treg, in SP compared with HD participants (ESM Fig. 3c).

Second, considering that the diminished suppression level in slow progressors could be attributed to either reduced suppressive ability of CD4^+^ Tregs or reduced responsiveness of CD4^+^ Tresponders to suppression in the co-culture, we performed allogeneic crossovers (Fig. 4e–h). We assessed the suppressive ability of Tregs in co-cultures, with HD responders and Tregs from the SP group. This revealed that SP Tregs demonstrated significantly reduced suppression of both proliferation and activation of the CD4^+^ Tresponders, at a 1:60 Treg:Tresponder ratio (*p* = 0.07; *p* < 0.05 respectively), compared with HD Tregs (Fig. 4f–g). Interestingly, this reduced suppression could be overcome with an increased Treg:Tresponder ratio, as demonstrated by no difference in suppression at the higher Treg:Tresponder ratios. This indicates that SP Tregs are impaired in their ability to suppress CD4^+^ T cells, although an increase in frequency may overcome this impaired suppression.

### Effector CD4^+^ T cells from slow progressors show increased responses to Treg suppression

**Fig. 5:**
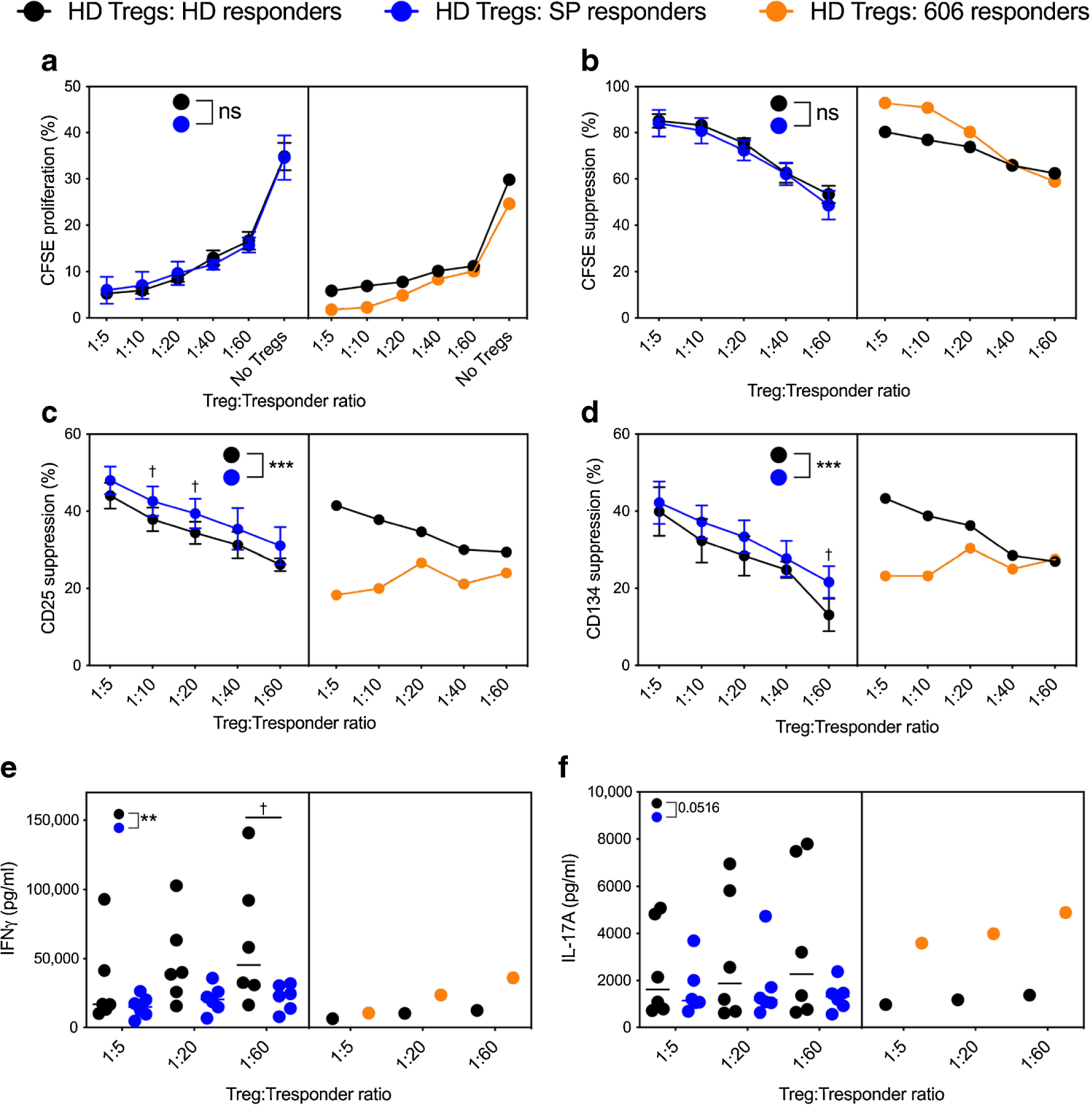
Effector CD4^+^ T cells are more responsive to suppression of T cell activation in slow progressors. The SP cohort is shown with blue lines and dots, the HD cohort with black lines and dots, and the orange line/dots represent donor SP 606. CD4^+^ T cells were sorted using a CD4^+^CD25^+^ Treg sorting kit. CD4^+^CD25^−^ (responders) were CFSE-labelled and Tregs were titrated at the observed ratios. Cells were activated with anti-CD3/28 beads and cultured for 3 days before flow cytometry (CD25 counterstain) and cytokine analysis. HD Tregs were cultured with either HD, SP or SP 606 responders. (**a**, **b**) CFSE proliferation (**a**) and percentage CFSE suppression (**b**). (**c**, **d**) Percentage suppression of CD25 (**c**) and CD134 (**d**). Percentage suppression was calculated using the positive control (activated responder cells with no Tregs). (**e**, **f**) IFNγ expression (**e**) and IL-17A (**f**) in co-cultures. ***p < 0.001, **p < 0.01, repeated measures two-way ANOVA. ^†^*p* < 0.05 Bonferroni’s post hoc multiple comparisons test

Third, we assessed CD4^+^ Tresponders and their resistance to suppression. We found that HD Tregs co-cultured with SP responders suppressed CD4^+^ T cell proliferation similarly to HD responders (Fig. 5a,b). However, suppression of CD25 and CD134 on SP CD4^+^ Tresponders was significantly increased, compared with HD (Fig. 5c,d). Strikingly, CD4^+^ Tresponders from SP 606 (orange line) were more resistant to suppression, as shown by CD25 (Fig. 5c) and CD134 measurement (Fig. 5d), compared with matched HD. To confirm these observations, we analysed proinflammatory cytokines IFNγ (Fig. 5e) and IL-17A (Fig. 5f). IFNγ expression was significantly reduced in co-cultures of HD Tregs with SP responders, compared with HD responders, at a 1:60 Treg:Tresponder ratio. IL-17A production in the co-cultures was variable; however, there was strikingly elevated IFNγ and IL-17A in the co-cultures with SP 606 responders (orange dots), compared with the matched HD. No significant differences in proinflammatory cytokines were observed when HD responders and SP Tregs were co-cultured (ESM Fig. 4a). In autologous co-cultures, we observed a modest decrease of IFNγ in each of the Treg:Tresponder ratios assessed, and again a more variable IL-17A production (ESM Fig. 4b). We found no differences in IL-2 concentration between the HD and SP donors (data not shown). Taken together, CD4^+^ Tregs from slow progressors showed impaired suppressive capacity towards effector CD4^+^ T cells; however, CD4^+^ effector T cells from slow progressors were more responsive to Treg-mediated suppression of T cell activation.

## Discussion

Our findings demonstrate that individuals who have been positive for multiple islet autoantibodies for 10 years or more, and have not developed overt type 1 diabetes, have a unique Treg signature. This signature is characterised by an increased frequency of activated memory Tregs enriched with GITR expression. However, surprisingly this is coupled with impaired Treg function, in terms of effector CD4^+^ T cell suppression. Conversely, effector CD4^+^ T cells in the slow progressors are more responsive to suppression.

Our study also features a unique snapshot of a slowly progressing individual (donor SP 606), assessed at a time of raised HbA_1c_, and this individual’s response was different from the others in the SP cohort. Therefore, we postulate that the mechanisms protecting slow progressors might be lost as they develop clinical symptoms of diabetes. Interestingly, no loss of Treg function was observed in this individual, but the effector CD4^+^ T cells were resistant to suppression of T cell activation, which was accompanied by an increase in IFNγ and IL-17 in Treg co-cultures. In terms of Treg numbers, this former slow progressor maintained the increase in Treg frequency but no enrichment of GITR expression. Interestingly, the level of GITR expression on the memory Treg_4 metacluster negatively correlated with HbA_1c_ levels measured in the SP cohort (data not shown), which should be noted for future studies.

Functional Treg studies in type 1 diabetes cohorts have demonstrated both impaired function in Tregs and a resistance to suppression in effector T cells. Together, both are likely to contribute to the impaired suppressive action, and a certain level of heterogeneity may exist in individuals with type 1 diabetes [6, 17]. We demonstrate impaired Treg function in parallel with an increased responsiveness to suppression in slow progressors. However, impaired Treg function in slow progressors could be overcome with an increase in the ratio of Tregs to effector T cells. This suggests that the increase in memory Tregs compensates for the impaired Treg function observed in the SP cohort, and the potency of Treg suppression is dose dependent. It is also interesting that, in the donor who had developed diabetes, this Treg impairment was not observed, supporting the notion that there is heterogeneity; however, we do not know if this individual had impaired Treg suppression before diagnosis.

Equally, we find the opposite pattern with effector T cells, with an increased response to suppression in slow progressors, which was specifically linked with effector T cell activation rather than proliferation, suggesting that Tregs can differentially regulate CD4^+^ T cell activation. The resistance to suppression in donor SP 606 was associated with an increase in proinflammatory cytokines, and therefore, it is possible that Tregs were responding to proinflammatory cytokines with increased activity. This is consonant with observations that, under certain circumstances, IFNγ is required for Treg-mediated suppression [18]. Furthermore, Tregs may also contribute to the increased IFNγ and IL-17A detected, as Tregs that are Th1- and Th17-like have been identified [19], and these Treg-produced proinflammatory cytokines may be required for suppressive activity [19, 20]. It should be noted that we did not observe any differences in IL-2 in Treg co-cultures (or in CD25 [IL-2Rα] expression), which would suggest that a lack of IL-2 production or competition for IL-2 was not involved in the Treg impairment. The mechanism for the increased sensitivity to suppression in the SP effector CD4^+^ T cells was not addressed in this study. However, we have previously demonstrated a decrease in CD95 expression on memory T cells [4], which may indicate an increased resistance to cell death. The increased response to suppression may be a global effector T cell feature in the SP cohort, or specific to the naive or memory subset, and, in our assays, the use of total effector CD4^+^CD25^−^ T cells is a limitation. Furthermore, Treg suppression assays in the presence of antigen presenting cells (dendritic cells) will provide further insight into slow progressor Treg function [21]. Overall, our study highlights the crucial interplay between Treg and effector T cells, and it will be important to understand the role and timing of Treg dysfunction during disease progression.

Unsupervised clustering revealed an increase in activated memory Tregs in slow progressors. Overall, Tregs in type 1 diabetes cohorts are similar in frequency to control individuals [17], although a reduction in activated FOXP3^+^ Tregs in type 1 diabetes has been reported [22]. Currently, we cannot determine whether the subsets we have identified represent Tregs at differing points in maturity or differentiation, or whether they are distinct subsets. Furthermore, additional Treg markers may be required for the identification of other Treg subsets [13] in this distinctive cohort. Future transcriptomic and functional studies on these Treg subsets would provide further insight, and it would be interesting to study the Treg gene signature in this SP group, which would provide a comparison with previous studies on both paediatric and adult new-onset type 1 diabetes cohorts [23, 24].

We observed an increase in the expression of GITR, a member of the TNF receptor (TNFR) family, in a distinct Treg metacluster, in slow progressors. GITR expression, which is frequently observed on activated Tregs [25] and is critical for suppressive action in Tregs, was shown by the use of anti-GITR antibodies [25, 26]. However, in some settings using anti-GITR agonistic antibodies has not affected the suppressive ability of Tregs, but rather induced proliferation and migration of pathogenic T cells [27]. Indeed, reduced expression of GITR on Tregs or a loss of GITR^+^ Tregs in individuals with type 1 diabetes does not result in an impaired suppressive phenotype, but the Tregs are more susceptible to apoptosis compared with those of healthy donors [28]. In mouse models, GITR is crucial for Treg expansion, as demonstrated both in *Gitr* (*Tnfrsf18*)-knockout mice, which have a reduced Treg frequency [29], as well as in *Gitr*-L (*Tnfsf18*)-deficient mice that demonstrate impaired Treg expansion [30]. These studies suggest that increased expression of GITR on Tregs in slow progressors may be the result of an increased propensity to develop or expand. Further functional studies are required to identify whether activated memory GITR^+^ Tregs in slow progressors have an improved survival mechanism or a resistance to apoptosis. Considering the importance of the GITR/GITR-L (GITR-ligand) pathway in both effector T cells and Tregs, it is also important to identify any difference in GITR-L signatures in immune cell compartments known to express this ligand.

A limitation in this study was the relatively small number of slow progressors available for study, but individuals followed for islet autoantibody positivity for up to 32 years are rare. In addition, age-matched individuals with new-onset type 1 diabetes were not included. It is a challenge to match our slow progressors to people with new-onset diabetes, as they were considerably older than many newly diagnosed individuals. For the future, it would be beneficial to replicate our findings in additional slow progressor cohorts, individuals with type 1 diabetes and individuals positive for multiple islet autoantibodies, with the latter allowing us to track and predict the individuals who will slowly progress. Our study describes a rare case of the islet autoantibody and Treg characteristics at diagnosis of type 1 diabetes in an older adult. Furthermore, we identify an immune cell signature in extreme slow progressors, and our study highlights the need to develop our understanding of Treg heterogeneity that exists during type 1 diabetes development, in order to help stratify those who would benefit from regulatory T cell therapy.

## Supplementary information

ESM (PDF 8762 kb)

## Data Availability

The datasets generated and/or analysed during the current study are available from the corresponding author on reasonable request.

## Abbreviations

BOX study: Bart’s Oxford study
CITRUS: Cluster identification, characterization, and regression
CTLA4: Cytotoxic T-lymphocyte-associated protein 4
FOXP3: Forkhead box P3
GADA: GAD autoantibodies
GITR: Glucocorticoid-induced TNFR-related protein
HD: Healthy donor (group)
IAA: Insulin autoantibodies
IA-2A: Islet antigen-2 autoantibodies
mAb: Monoclonal antibody
MSD: Meso Scale Discovery
MST: Minimum spanning tree
PBMC: Peripheral blood mononuclear cell
SP: Slow progressor (group)
Treg: Regulatory T cell
Tresponder: Responder T cell
t-SNE: t-distributed stochastic neighbour embedding
ZnT8A: Zinc transporter 8 autoantibodies

## References

[1] Ziegler AG, Rewers M, Simell O (2013). Seroconversion to multiple islet autoantibodies and risk of progression to diabetes in children. JAMA.

[2] Insel RA, Dunne JL, Atkinson MA (2015). Staging presymptomatic type 1 diabetes: a scientific statement of JDRF, the Endocrine Society, and the American Diabetes Association. Diabetes Care.

[3] Long AE, Wilson IV, Becker DJ (2018). Characteristics of slow progression to diabetes in multiple islet autoantibody-positive individuals from five longitudinal cohorts: the SNAIL study. Diabetologia.

[4] Hanna SJ, Powell WE, Long AE (2020). Slow progressors to type 1 diabetes lose islet autoantibodies over time, have few islet antigen-specific CD8. Diabetologia.

[5] Long SA, Cerosaletti K, Bollyky PL (2010). Defects in IL-2R signaling contribute to diminished maintenance of FOXP3 expression in CD4(+)CD25(+) regulatory T-cells of type 1 diabetic subjects. Diabetes.

[6] Schneider A, Rieck M, Sanda S, Pihoker C, Greenbaum C, Buckner JH (2008). The effector T cells of diabetic subjects are resistant to regulation via CD4+ FOXP3+ regulatory T cells. J Immunol.

[7] Garg G, Tyler JR, Yang JH (2012). Type 1 diabetes-associated IL2RA variation lowers IL-2 signaling and contributes to diminished CD4+CD25+ regulatory T cell function. J Immunol.

[8] Bingley PJ, Gale EA (1989). Incidence of insulin dependent diabetes in England: a study in the Oxford region, 1985-6. BMJ.

[9] Long AE, Gillespie KM, Rokni S, Bingley PJ, Williams AJ (2012). Rising incidence of type 1 diabetes is associated with altered immunophenotype at diagnosis. Diabetes.

[10] Bonifacio E, Yu L, Williams AK (2010). Harmonization of glutamic acid decarboxylase and islet antigen-2 autoantibody assays for national institute of diabetes and digestive and kidney diseases consortia. J Clin Endocrinol Metab.

[11] Van Gassen S, Callebaut B, Van Helden MJ (2015). FlowSOM: using self-organizing maps for visualization and interpretation of cytometry data. Cytometry A.

[12] Kotecha N, Krutzik PO, Irish JM (2010). Web-based analysis and publication of flow cytometry experiments. Curr Protoc Cytom Chapter.

[13] Mason GM, Lowe K, Melchiotti R (2015). Phenotypic complexity of the human regulatory T cell compartment revealed by mass cytometry. J Immunol.

[14] Brusko TM, Wasserfall CH, Clare-Salzler MJ, Schatz DA, Atkinson MA (2005). Functional defects and the influence of age on the frequency of CD4+ CD25+ T-cells in type 1 diabetes. Diabetes.

[15] Polikowsky HG, Drake KA (2019). Supervised machine learning with CITRUS for single cell biomarker discovery. Methods Mol Biol.

[16] Long AE, Tatum M, Mikacenic C, Buckner JH (2017). A novel and rapid method to quantify Treg mediated suppression of CD4 T cells. J Immunol Methods.

[17] Lawson JM, Tremble J, Dayan C (2008). Increased resistance to CD4+CD25hi regulatory T cell-mediated suppression in patients with type 1 diabetes. Clin Exp Immunol.

[18] Mori Y, Kodaka T, Kato T, Kanagawa EM, Kanagawa O (2009). Critical role of IFN-gamma in CFA-mediated protection of NOD mice from diabetes development. Int Immunol.

[19] Duhen T, Duhen R, Lanzavecchia A, Sallusto F, Campbell DJ (2012). Functionally distinct subsets of human FOXP3+ Treg cells that phenotypically mirror effector Th cells. Blood.

[20] McClymont SA, Putnam AL, Lee MR (2011). Plasticity of human regulatory T cells in healthy subjects and patients with type 1 diabetes. J Immunol.

[21] Shevach EM (2009). Mechanisms of foxp3+ T regulatory cell-mediated suppression. Immunity.

[22] Okubo Y, Torrey H, Butterworth J, Zheng H, Faustman DL (2016). Treg activation defect in type 1 diabetes: correction with TNFR2 agonism. Clin Transl Immunol.

[23] Pesenacker AM, Wang AY, Singh A (2016). A regulatory T-cell gene signature is a specific and sensitive biomarker to identify children with new-onset type 1 diabetes. Diabetes.

[24] Pesenacker AM, Chen V, Gillies J (2019). Treg gene signatures predict and measure type 1 diabetes trajectory. JCI Insight.

[25] McHugh RS, Whitters MJ, Piccirillo CA (2002). CD4(+)CD25(+) immunoregulatory T cells: gene expression analysis reveals a functional role for the glucocorticoid-induced TNF receptor. Immunity.

[26] Shimizu J, Yamazaki S, Takahashi T, Ishida Y, Sakaguchi S (2002). Stimulation of CD25(+)CD4(+) regulatory T cells through GITR breaks immunological self-tolerance. Nat Immunol.

[27] You S, Poulton L, Cobbold S (2009). Key role of the GITR/GITRLigand pathway in the development of murine autoimmune diabetes: a potential therapeutic target. PLoS One.

[28] Xufré C, Costa M, Roura-Mir C (2013). Low frequency of GITR+ T cells in ex vivo and in vitro expanded Treg cells from type 1 diabetic patients. Int Immunol.

[29] Ronchetti S, Nocentini G, Riccardi C, Pandolfi PP (2002). Role of GITR in activation response of T lymphocytes. Blood.

[30] Liao G, O'Keeffe MS, Wang G (2014). Glucocorticoid-induced TNF receptor family-related protein ligand is requisite for optimal functioning of regulatory CD4(+) T cells. Front Immunol.

