# Supplementary material for: Activated but functionally impaired memory Tregs are expanded in slow progressors to type 1 diabetes
Source: Diabetologia. 2021 Oct 28;65(2):343–55. doi: 10.1007/s00125-021-05595-0 (PMC8741669; doi:10.1007/s00125-021-05595-0)
Supplement: Supplementary file 1 — (PDF 8762 kb) [file 125_2021_5595_MOESM1_ESM.pdf]

ID	Sex	Age at current sample (years)	Time since mAab+ve (years)	Current GADA DK Units/ml	Current IA-2A DK units/ml	Current IAA units	Current ZnT8A units	Antibodies present at first mAab+ve sample	Stage of Diabetes Progression
SP 606	Male	72.6	32	53.929	0.000	0.000	34.525	GADA, IA-2A, ZnT8A	Stage 3
SP 607	Male	49.1	30.8	34.323	0.000	0.000	0.851	GADA, ZnT8A	Stage 1
SP 608	Female	47.1	30.4	176.307	23.100	0.000	3.862	GADA, IA-2A, ZnT8A	Stage 2
SP 609	Male	33.5	26.2	114.170	0.275	0.000	1.481	GADA, IAA	Stage 1
SP 610	Female	40.6	25.6	4.524	35.430	0.000	0.361	GADA, IA-2A, ZnT8A	Stage 1
SP 611	Male	56.3	18.6	1026.042	4.791	1.640	0.435	GADA, IAA	Stage 1
SP 612	Male	31.2	18.2	319.394	0.000	0.000	0.312	GADA, IAA	Stage 1
SP 613	Male	31.7	18.6	143.477	6.748	0.000	4.670	GADA, IA-2A, ZnT8A	Stage 1

ESM Table 1. Current islet autoantibody results for 8 slow progressors. The positive thresholds for GADA are >=33 DK units/ml, IA-2A are >=1.4 DK units/ml, IAA are >=0.2 units and ZnT8 are >=1.8 units.

ID	DRB1 (allele 1)	DRB1 (allele 2)	DQB1 (allele 1)	DQB1 (allele 2)	Genotype	Level of genetic risk
SP 606	04	12	0301	0302	DR4-DQ8/X	Moderate
SP 607	15	04	0302	0601	DR4-DQ8/X	Moderate
SP 608	04	11	0301	0302	DR4-DQ8/X	Moderate
SP 609	03	04	0201	0302	DR3-DQ2/DR4-DQ8	High
SP 610	04	04	0301	0302	DR4-DQ8/DR4-DQ7	Moderate
SP 611	04	07	02	0302	DR4-DQ8/X	Moderate
SP 612	01	03	02	0501	DR3-DQ2/X	Moderate
SP 613	03	04	0201	0302	DR3-DQ2/DR4-DQ8	High

ESM Table 2. Table of genetic information for 8 slow progressors.

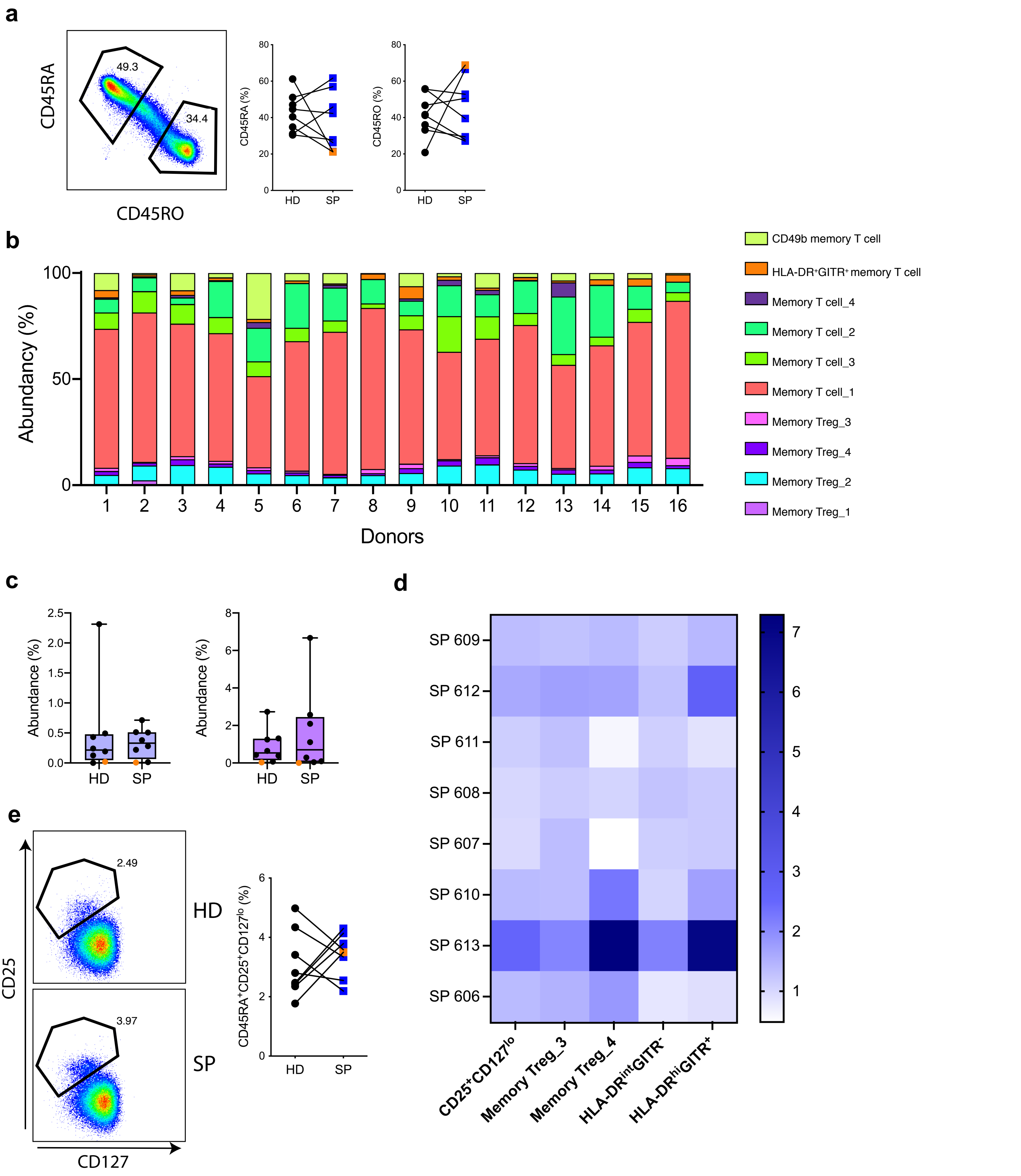

ESM Figure 1. *Treg metaclusters in slow progressors.* (a) Gating (left) and summary graphs for CD45RA (middle) and CD45RO (right) $CD4^+$ T cells in slow progressors, matched to healthy donors. (b) $CD45RA^-$ Treg metaclusters (Memory T cell_1; Memory T cell_2; Memory T cell_3; Memory T cell_4; CD49b memory T cell; $HLA\text{-}DR^+GITR^+$ memory T cell; Memory Treg_1; Memory Treg_2; Memory Treg_3; Memory Treg_4) defined in each donor (n=16). (c) Memory Treg_1 (left) and Memory T cell_4 (right) box-plots (mean <0.05%) (d) Heatmap showing expression ratio (SP/HD) of defined Treg metaclusters Memory Treg_3 and Memory Treg_4 along with hierarchical gating emulating both Treg metaclusters for each individual slow progressor donor. (e) Gating to show $CD25^+CD127^{lo}$ Treg cells in the CD45RA $CD4^+$ T cell compartment and summary graph showing no difference in naive Tregs.

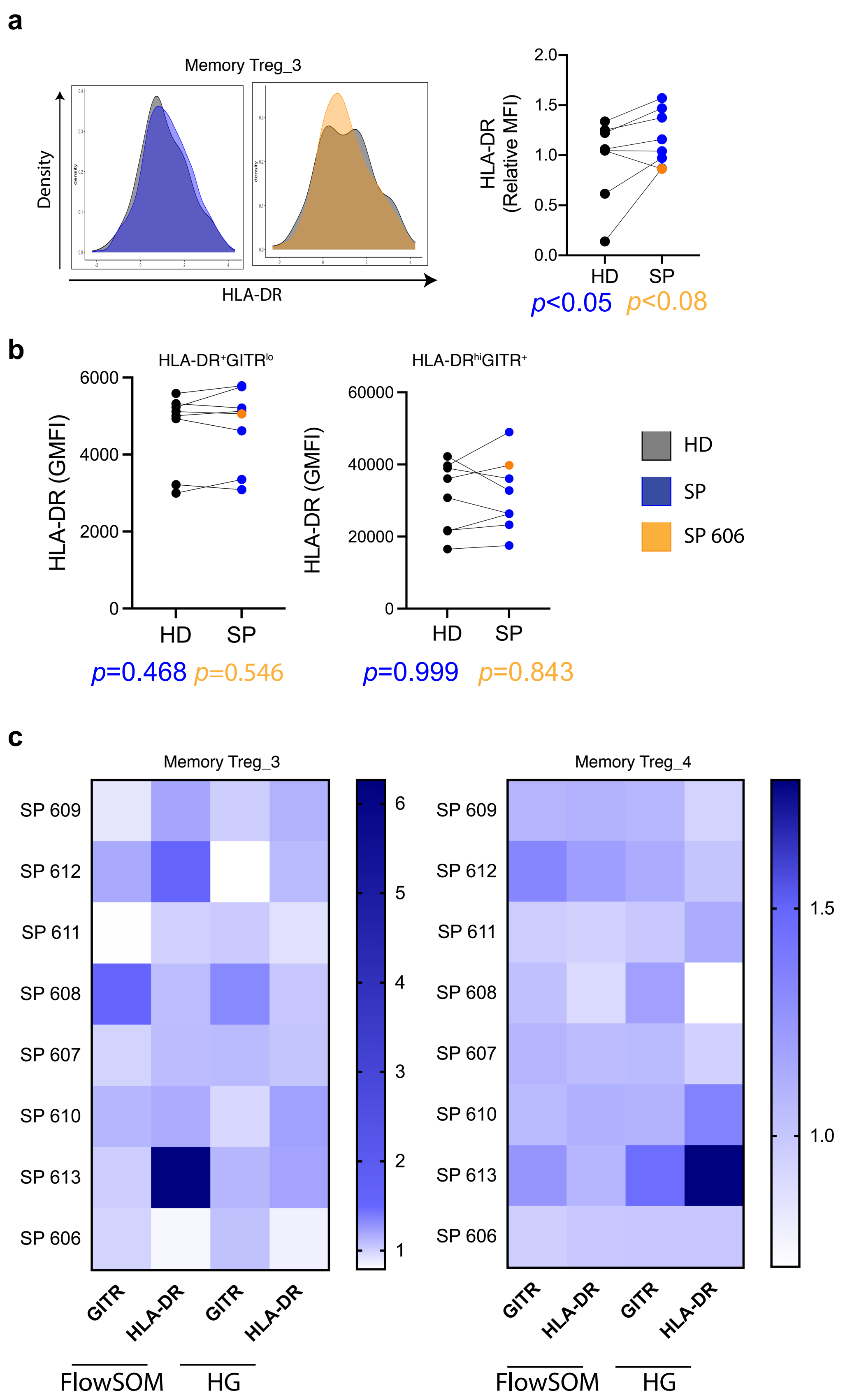

ESM Figure 2. *HLA-DR expression in Memory Treg_3 and Memory Treg_4 metaclusters.* Memory Treg_3 metacluster from FLOWSOM, was examined for a change in HLA-DR expression marker. (a) FlowSOM HLA-DR expression concatenated from all HD (grey), all SP (blue) and SP 606 (orange) showing histograms (left) and summary graph (right). (b) Summary graphs of HLA-DR expression, from hierarchical gating in both HLA-DR^{+}GITR^{lo} and HLA-DR^{hi}GITR^{+} Tregs (a, b) Wilcoxon matched-pairs signed rank test, *p*-value in orange is all donors included, *p*-value in blue is without donor SP 606 and matched HD not included in test. (c) Heatmap showing expression GITR and HLA-DR ratios (SP/HD), for each individual slow progressor donor, in defined Treg metaclusters or subsets defined by hierarchical gating: Memory Treg_3 (HLA-DR^{+}GITR^{lo}) and Memory Treg_4 (HLA-DR^{hi}GITR^{+}).

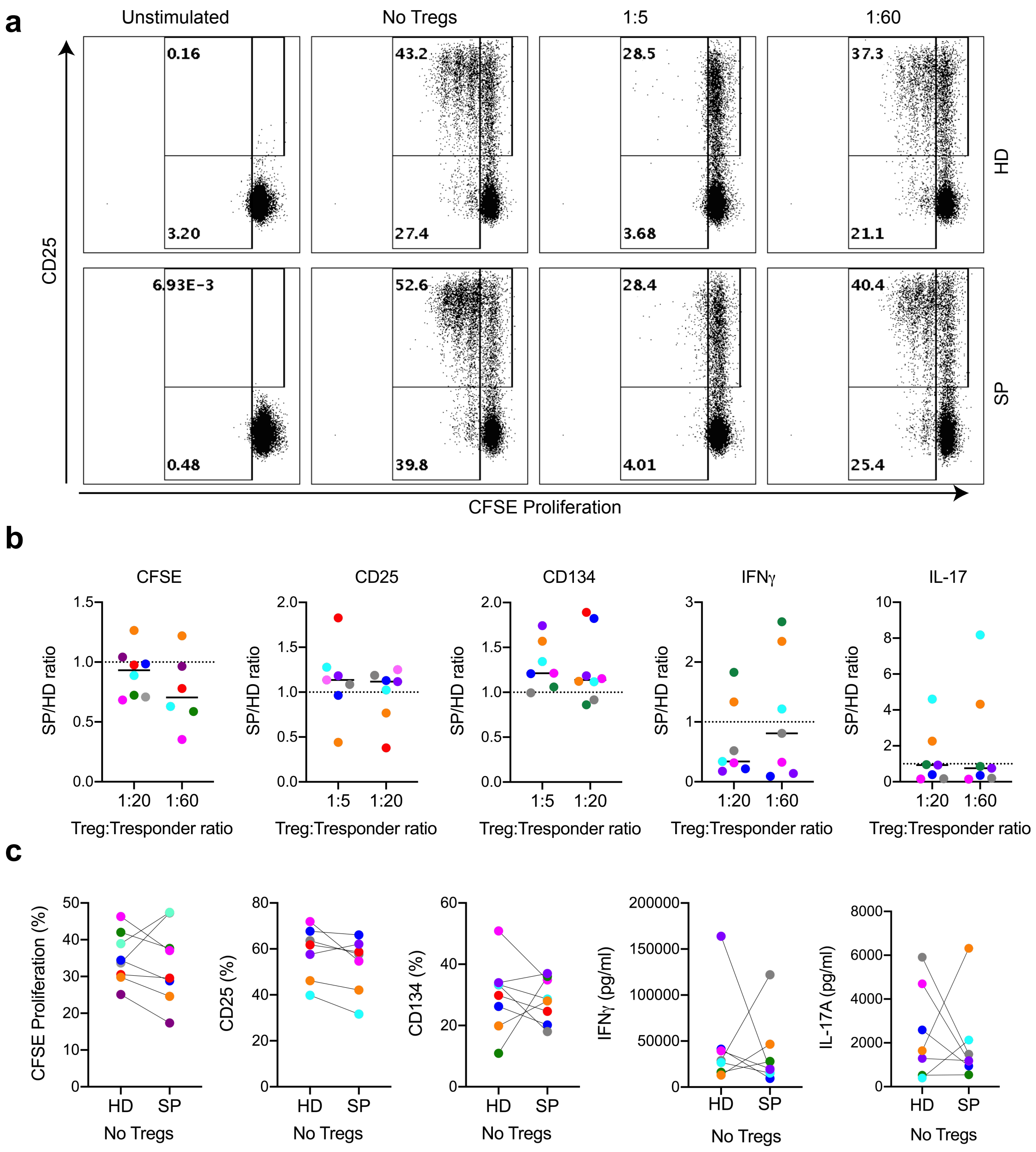

ESM Figure 3. *Treg suppression in slow progressors* (a) Representative flow plots for Treg suppression assay showing CFSE and CD25 gating on both slow progressor donor (SP) and matched healthy donor (HD). Unstimulated control, no Tregs (positive control) Treg:responder ratios 1:5 and 1:60 are shown (b) SP/HD ratios for CFSE, CD25, CD134, IFNγ and IL-17 when autologous Tregs and responders are cultured. (c) Response of CD4 effector T cells to polyclonal stimuli anti-CD3/28 for each individual slow progressor donor matched to healthy controls.

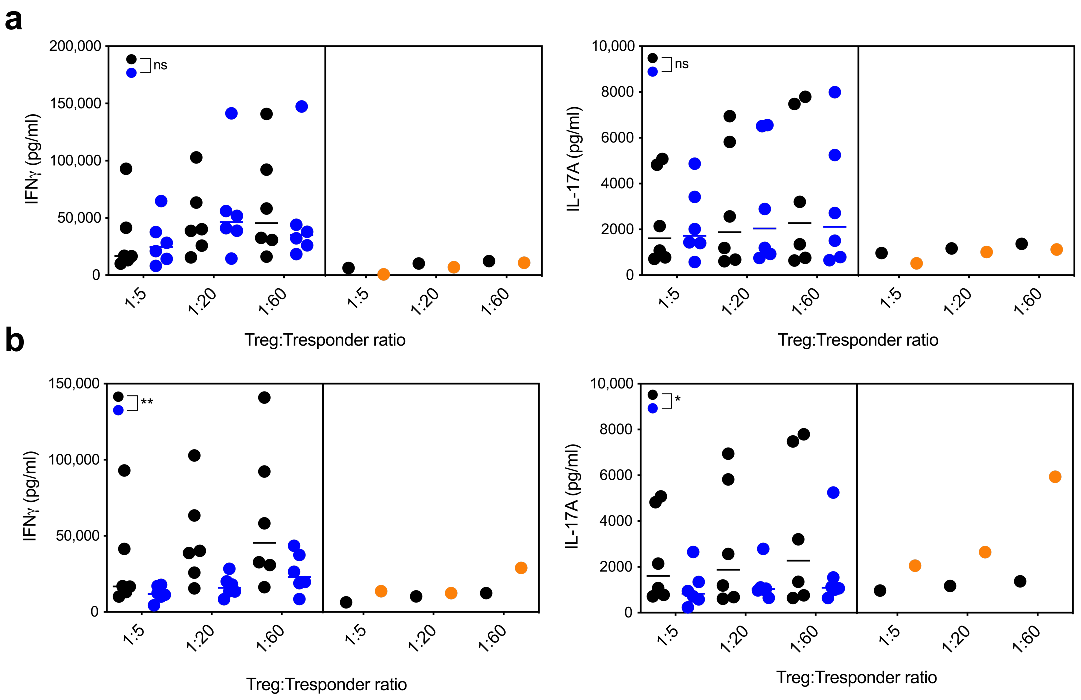

ESM Figure 4. *Proinflammatory cytokine responses in Treg suppression assay.* (a) HD responders were co-cultured with Tregs from matched healthy donors and slow progressors or SP 606. (b) autologous Treg: T responder assays (HD Tregs:HD responders; SP Tregs:SP responders). IFNγ (left) and IL-17 (right) production measured by Meso Scale Discovery. Healthy donor (HD, black dots), HD 606 plotted separately and slow progressors (SP, blue dots) or SP 606 donor (orange dots). ns, not-significant,**<0.01, *<0.05, repeated measures-two-way ANOVA.
